# Nanomaterials for Allergy Diagnosis and Treatment: Advances, Opportunities and Translational Challenges

**DOI:** 10.1002/advs.76169

**Published:** 2026-06-22

**Authors:** Madiha Habib, Shan Jiang, Joyce ZX Lee, CW Lim, HL Yeung, Nicki YH Leung, Patrick SC Leung, Ting F Leung, Christine YY Wai

**Affiliations:** ^1^ Department of Paediatrics The Chinese University of Hong Kong Hong Kong SAR China; ^2^ Faculty of Medicine The Chinese University of Hong Kong Hong Kong SAR China; ^3^ Division of Life Science The Hong Kong University of Science and Technology Hong Kong SAR China; ^4^ Division of Rheumatology Allergy and Clinical Immunology University of California Davis California USA; ^5^ Hong Kong Hub of Paediatric Excellence The Chinese University of Hong Kong Hong Kong SAR China

**Keywords:** allergic diseases, diagnosis, immunotherapy, nanomaterials, nanovaccine

## Abstract

Allergic disorders, including food allergy, asthma, and atopic dermatitis, affect an estimated 10–30% of the global population, with prevalence continuing to rise in industrialized countries. Allergy is driven by dysregulated type 2 T‐helper cells (Th2) and immunoglobulin E (IgE) antibody responses. A range of diagnostic tools is available, but most methods are limited by variable sensitivity and specificity, and the inability to predict clinical reactivity. Although allergen‐specific immunotherapy (AIT) remains the only etiological therapeutic method for allergic disorders, conventional AITs are limited by frequent administration, risk of adverse events, suboptimal patient adherence, and inconsistent long‐term efficacy. Advances in nanotechnology offer emerging opportunities for improving allergy diagnosis and treatment through enhanced analytical sensitivity, targeted allergen delivery and controlled immune modulation. This review provides a comprehensive overview of the latest research on nanomaterials, including their application in nanomaterials‐based diagnostic systems and nano‐enabled immunotherapies. We highlight their roles in improving allergen‐specific IgE detection, refining functional cellular assays, and enabling next‐generation immunotherapies through controlled allergen delivery and immunomodulation. We also critically examine key translational barriers and outline essential future directions required for translating nanotechnologies into clinical practice in allergy medicine.

## Graphical abstract

Nanomaterials offer dual applications in allergy management. For diagnosis, nanomaterials enhance analytical sensitivity and improve detection in specific IgE and functional assays such as the basophil activation test (BAT). For allergen‐specific immunotherapy, nanomaterials enable allergen masking and controlled release, and effectively modulate the immune response.

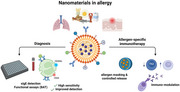

**Abbreviations**
ACB NGs: Ag_2_S–CeO_2_@BSA–AB nanohybrids
AgNPs: Silver nanoparticles
AIT: Allergen‐specific immunotherapy
AK: Arginine kinase
APCs: Antigen‐presenting cells
AuNPs: Gold nanoparticles
BAT: Basophil activation test
CdTe: Cadmium telluride
CL: Chemiluminescence
CuMVTT: Cucumber Mosaic Virus integrated with universal T‐cell epitopes
CUR: Curcumin
CVD: Chemical vapour deposition
DCs: Dendritic cells
DLS: Dynamic light scattering
DNP: 2,4‐dinitrophenyl
DONs: DNA origami nanostructures
EMA: European Medicines Agency
EPIT: Epicutaneous immunotherapy
FA: Food allergy
FDA: Food and Drug Administration
FRET: Fluorescence resonance energy transfer
FTIR: Fourier‐transform infrared spectroscopy
HDM: House dust mite
HRP: Horseradish peroxidase
Ig: Immunoglobulin
IL: Interleukin
InP/ZnS: Indium phosphide (InP) core encapsulated by a zinc sulfide (ZnS) shell
LNPs: Lipid‐based nanoparticles
LOD: Limit of detection
MAT: Mast cell activation test
MEF: Metal‐enhanced fluorescence
Ni‐NTA: Nickel‐nitrilotriacetic acid
NPs: Nanoparticles
OFC: Oral food challenge
OIT: Oral immunotherapy
o‐PD: o‐phenylenediamine
OVA: Ovalbumin
PAMAM: Polyamidoamine
PEG: Polyethylene glycol
PLGA: Poly(lactic‐co‐glycolic acid)
QB‐FLISA: Quantum dot nanobead‐based fluorescence‐linked immunosorbent assay
QDs: Quantum dots
SCIT: Subcutaneous immunotherapy
SGB: Stellate ganglion block
sIgE: specific immunoglobulin E
SiNPs: Silica nanoparticles
SLIT: Sublingual immunotherapy
SPT: Skin prick test
TEM: Transmission electron microscopy
Tfh: Follicular helper T cells
TGA: Thermogravimetric analysis
Th2: Type 2 T‐helper cells
Tregs: Regulatory T cells
VLPs: Virus‐like particles
XPS: X‐ray photoelectron spectroscopy

## 1 Introduction

Over the past few decades, the prevalence of allergic diseases, including respiratory allergies [1, 2], food allergy (FA) [3], asthma [4] and atopic dermatitis [5], has risen significantly. The number of affected individuals has increased markedly in industrialized countries, now impacting over 20–30% of the population around the world [6, 7]. Fundamentally, allergy is a disorder of the immune system characterized by hypersensitivity reactions to typically harmless antigens, termed allergens. These allergens may originate from the environment (inhalant allergens), food, medications or insect bites. Allergic conditions exert a substantial burden on patients’ quality of life and impose considerable financial strain on healthcare systems [8], underscoring the critical need for advancements in both diagnostic and therapeutic approaches to improve patient outcomes.

### 1.1 Basic Pathology of Allergy

**FIGURE 1:**
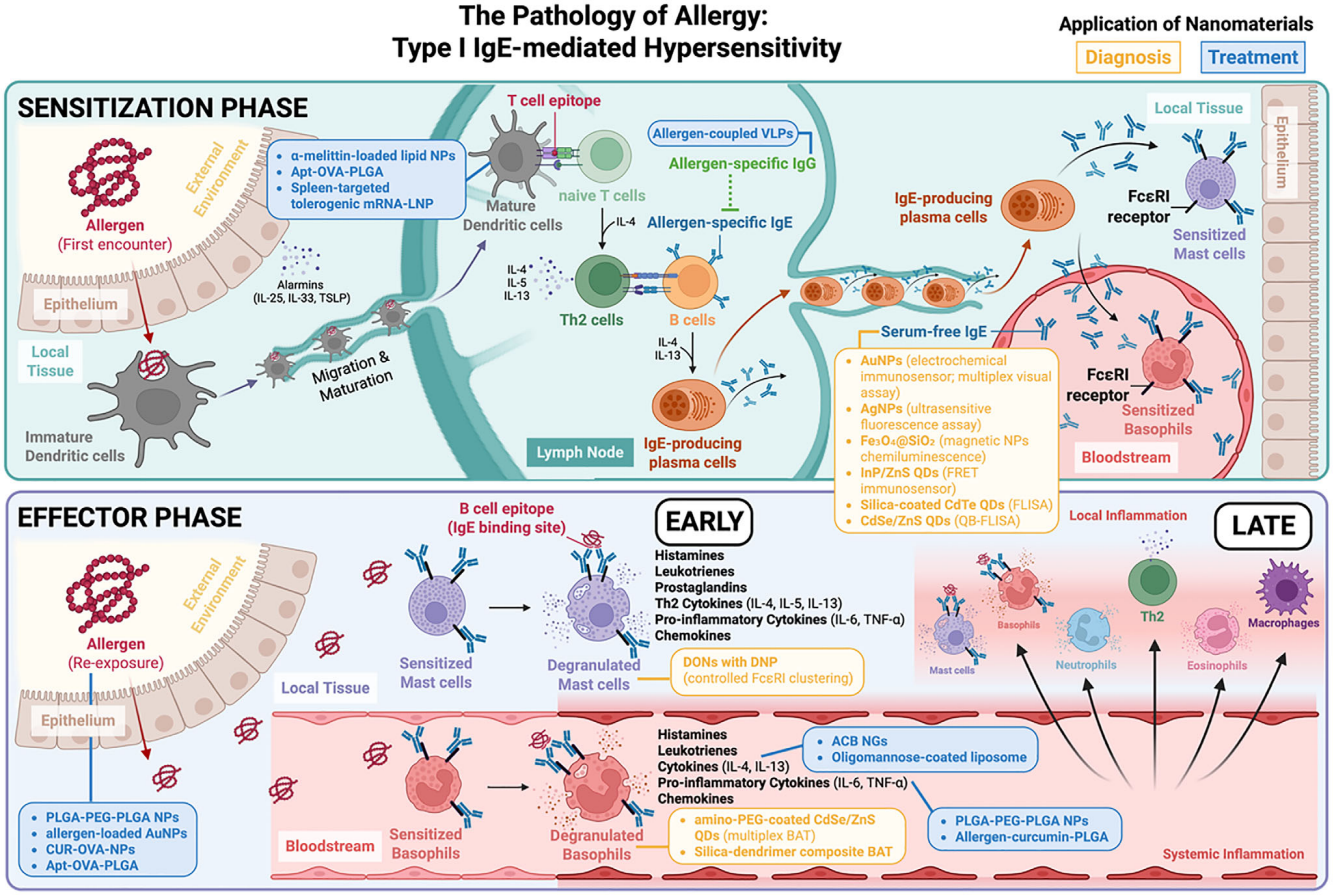
The pathology of allergy, divided into sensitization and effector phases, and how nanomaterials can provide a novel approach in allergy diagnosis and treatment.

While the clinical and pathological manifestations of different allergic diseases vary, the allergy cascade typically involves sensitization followed by early‐ and late‐phase reactions (Figure 1), predominantly mediated by the production of allergen‐specific immunoglobulin E (IgE). IgE production is initiated when dendritic cells (DCs) process and present allergen epitopes to naïve T‐helper cells, promoting their differentiation into type 2 T helper (Th2) cells. Activated Th2 cells then drive the isotype switch of B cells from IgG‐ and IgM‐producing to synthesizing allergen‐specific IgE through Th2 cytokines, including interleukin (IL)‐4, IL‐5, and IL‐13, thereby establishing “sensitization” [9]. Recent evidence suggests that follicular helper T cells (Tfh) may play an even more prominent role than Th2 cells in facilitating IgE class switching in germinal centers, providing new insights into the mechanisms underlying allergic sensitization [10]. Upon re‐exposure to the same allergen, cross‐linking of IgE on FcεRI receptors of mast cells and basophils leads to cellular activation and degranulation, releasing inflammatory mediators such as histamine, leukotrienes, and prostaglandins, which drive early‐phase reaction and acute allergic symptoms ranging from mild to severe, including pruritus, sneezing, hives, bronchospasms and even life‐threatening circulatory collapses seen in systemic anaphylaxis [11]. Sensitized individuals may also experience cross‐reactivity, reacting to structurally similar allergens from different sources. This can complicate allergy diagnosis and management, reinforcing the importance of molecular‐level allergen characterization.

### 1.2 Allergy Diagnosis

Current diagnostic methods for IgE‐mediated type 1 (immediate) allergies include the skin prick test (SPT), serum specific IgE (sIgE) testing, basophil activation test (BAT), mast cell activation test (MAT), provocation test, and others. SPT remains the first‐line in vivo method, as it is widely available, minimally invasive, and provides immediate results (in 15–20 min) to confirm sensitization. This method involves introducing small quantities of specific allergens into the skin, which causes histamine release when IgE‐allergen cross‐linking occurs on mast cell receptors. When the subject is allergic to the specific allergen, a wheal‐and‐flare response is generated, which can offer results in a semi‐quantitative manner [12]. In vitro sIgE quantification measures the level of circulating IgE specific to an allergen extract. sIgE tests are unaffected by antihistamine drugs and are often useful, especially when SPT cannot be performed [13]. Although SPT and specific IgE assays usually had high sensitivity [14], they only reflect sensitization in the absence of true clinical allergy and suffer from variable sensitivity/specificity and cross‐reactivity between allergen sources [15]. Test panels often miss relevant or regional allergens [16, 17]. Accurate diagnosis depends heavily on precise clinical history and timing, while standardized extracts and cutoff values vary across laboratories. Furthermore, neither method reliably predicts reaction severity, threshold doses for clinical reactivity, or long‐term prognosis, nor do they consistently identify patients who will benefit from allergen‐specific immunotherapy (AIT) [18].

With the advancement in the molecular identification and cloning of allergens and their epitopes, component‐resolved diagnosis (CRD), which quantifies IgE to specific allergens instead of crude extracts, improves the specificity of food allergy diagnosis [15]. For example, measuring specific IgE to Ara h 2 for peanut allergy demonstrated test specificity at 98%, compared with the specificity at 86% for peanut extract‐specific IgE measurement [19]. Beyond protein‐based IgE quantification, epitope‐specific IgE antibody testing further minimize irrelevant signals from cross‐reactive IgE antibodies. IgE binding to epitopes was found to be independent of patients’ IgE levels to the respective allergen [20]. Apart from improving diagnostic accuracy, epitope‐based diagnostics are predictive of peanut‐allergic patients’ cumulative tolerated thresholds and could stratify peanut‐allergic patients into high‐ and low‐threshold groups [21].

Functional cell‐based assays like basophil activation test (BAT) and mast cell activation test (MAT) also allow improved specificity and better discrimination between true sensitization and cross‐reactivity [15]. BAT and MAT are in vitro flow cytometry‐based assays, where activation markers such as CD63 and CD203c (BAT) and CD107 (MAT) are upregulated on the surface of basophils or mast cells after allergen *ex vivo* stimulation. BAT had high specificity to support the diagnosis of food allergy [14]. However, both assays are limited by the lack of broad standardization, restricted availability, and high operation costs [13, 22]. Ultimately, the oral food challenge (OFC) remains the gold standard for diagnosing FA. OFC is an in vivo test performed under supervised conditions; the subject is guided to ingest gradually increasing amounts of the suspected food allergen. Despite its diagnostic value, this procedure can provoke significant patient anxiety due to the risk of inducing severe allergic reactions, including anaphylaxis [23]. These highlight the current unmet need for more effective and accurate diagnostics for allergy disorders.

### 1.3 Allergen‐Specific Immunotherapy

Allergen‐specific immunotherapy is a disease‐modifying treatment for IgE‐mediated allergic disorders that induces clinical tolerance by the repeated administration of increasing amounts of the causal allergen [24]. Delivered subcutaneously (SCIT) or sublingually (SLIT), orally (OIT), or epicutaneously (EPIT) [25], AIT can induce DCs with a pro‐regulatory phenotype, shift immune responses from Th2/IgE‐dominant profiles toward regulatory T cells, IgG4 and/or IgA‐mediated pathways, thus reducing symptoms and preventing disease progression [26]. Typical regimens run 3–5 years for lasting benefit. But AITs are not without risks; common side effects include local reactions, systemic or anaphylactic reactions, so patient selection and continuous monitoring throughout treatment are essential [27].

SCIT involves regular injections of gradually increasing doses of a standardized allergen extract into the subcutaneous tissue, while SLIT delivers allergen extracts as drops or tablets held under the tongue daily, allowing mucosal uptake and immune modulation. Both routes of AIT have confirmed efficacy for seasonal rhinitis and rhinitis due to house dust mite (HDM), grass, ragweed, birch, and Japanese cedar pollen allergens, although a long treatment for 3 years is generally necessary to maintain tolerance after stopping therapy [28, 29]. The two forms of AIT also have distinct mechanisms of action – SCIT mediates its IgE‐inhibitory activity by IgG4, whereas SLIT often induces IgA1 and/or IgA2 [30].

While AIT for inhalant allergies is comparatively more well‐established, immunotherapy for FA remains more limited, with most studies focused on peanut allergy. OIT involves daily ingestion of gradually escalating doses of peanut proteins to increase the threshold for clinical reactivity and reduce risk from accidental exposures. OIT has shown robust desensitization for peanut allergy with increased threshold dose achieved in 67.2% of patients compared to only 4% who received placebo [31]. However, OIT comes with a high rate of side effects, including systemic allergic side effects that occurred in 14.2% of patients, including severe anaphylaxis, thus requiring careful dose escalation and maintenance. Peanut OIT is currently not recommended outside specialist centres. EPIT uses allergen‐containing patches applied to intact skin to stimulate resident DCs and induce immune tolerance. Peanut EPIT has a low risk of systemic reactions, with common local skin irritation at the patch site; efficacy tends to be modest compared with oral routes (35.3% achieved desensitization) but may offer a safer alternative for certain patients, especially children [32]. However, FA immunotherapy has shown limited evidence of durable, long‐term efficacy after discontinuation [33], underscoring the need for ongoing maintenance dosing and individualized risk‐benefit assessment.

Given the limitations of current AIT approaches and the rapid advancement in molecular technologies, more targeted and safer strategies are under active investigation. These include molecular diagnosis‐guided allergen selection, engineered hypoallergenic variants, DNA‐based vaccines, allergen combinations and the incorporation of nanomaterials with immunomodulatory properties to enhance AIT safety and therapeutic precision [26]. These innovations aim to create more effective, safer, and personalized AIT regimens for the next generation of clinical practice.

## 2 Nanomaterials

There has been growing interest in nanotechnology as a promising alternative for allergy management [34]. Nanotechnology involves research, engineering, and technology at the nanoscale (approximately 1–100 nm), where materials exhibit properties markedly distinct from their bulk counterparts and offer diverse biomedical applications [8]. Their small size provides them with distinctive properties such as enhanced reactivity, unique optical characteristics, and improved mechanical strength resulting from quantum confinement and surface effects [35]. Nanomaterials also possess an exceptionally large surface‐area‐to‐volume ratio that enables high drug‐loading capacity and extended circulation time within the vascular system. Nanotechnology is a novel approach enabling more targeted and effective diagnostic and therapeutic applications in allergy [34].

### 2.1 Types of Nanomaterials

**FIGURE 2:**
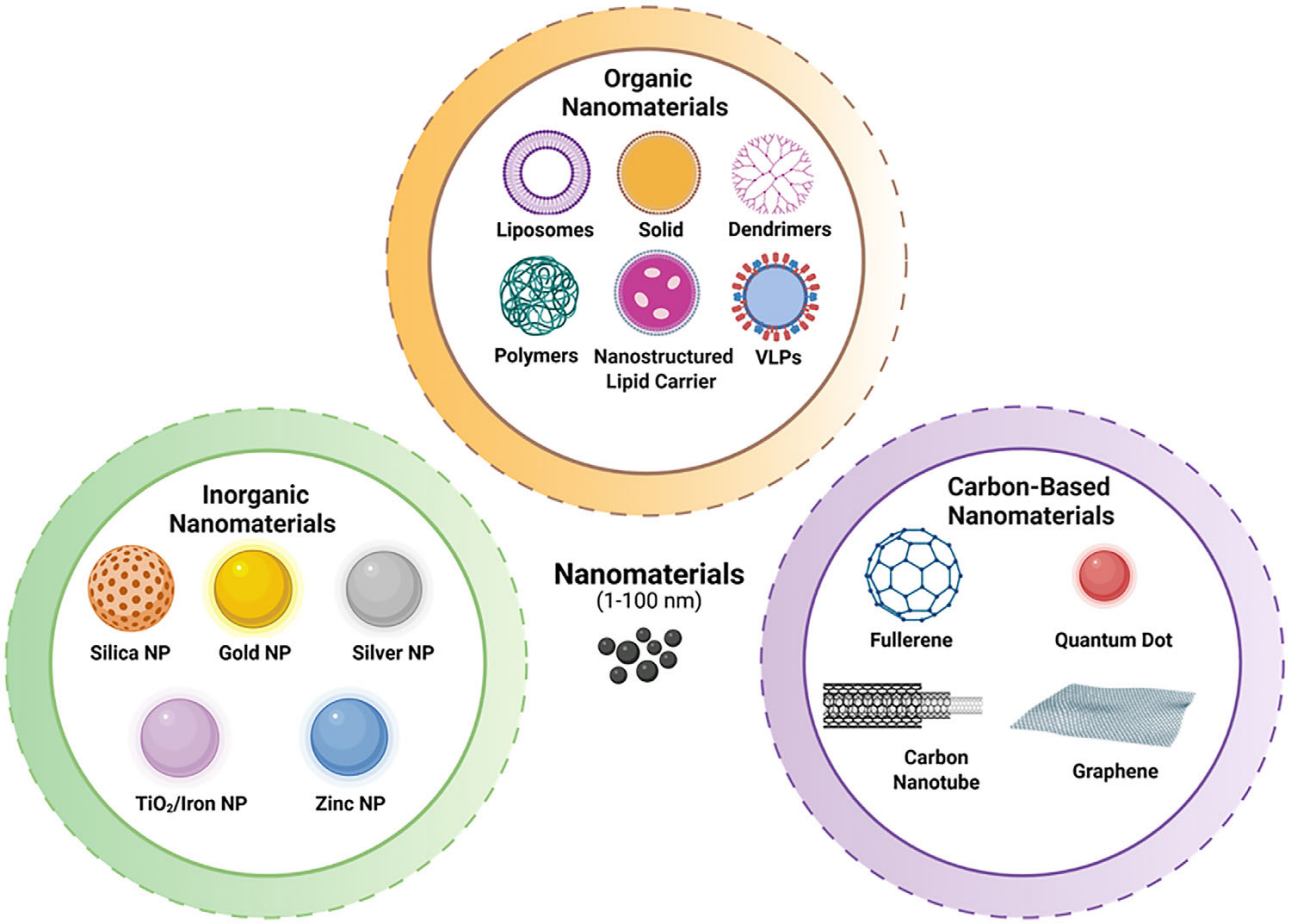
Types of nanomaterials including organic, inorganic, and carbon‐based nanomaterials.

Depending on their chemical makeup as depicted in Figure 2, nanomaterials employed in biomedical applications are broadly classified into three main categories: organic, inorganic, and carbon‐based nanoparticles [9].

1. *Organic nanomaterials*. Organic nanomaterials are composed of biologically derived components, including proteins, lipids, carbohydrates, and biodegradable polymers [36, 37]. Common examples include virus‐like particles (VLPs), polymers, liposomes, dendrimers, micelles, nanocapsules, and ferritin, which are all widely used in biomedical research and applications. These nanomaterials are often engineered to dimensions under 100 nm and exhibit excellent biocompatibility. They are non‐toxic, degradable formulations with hollow cores and are responsive to external triggers like thermal changes, electromagnetic fields, or photonic irradiation [38]. These features render them highly suitable as transporters for drug molecules and genetic materials [39].
2. *Inorganic nanomaterials*. Inorganic nanoparticles include metal salts, metal oxides, and ceramics. These particles exhibit excellent chemical stability, aqueous solubility, and advantageous biocompatibility, offering enhanced control over physicochemical parameters and greater resistance to degradation as compared to organic nanoparticles [40]. Ceramic nanoparticles are non‐metallic inorganic solids predominantly made of oxides, carbides, carbonates, or phosphates, often synthesized via high‐temperature processing and subsequent controlled cooling. Ceramic nanoparticles have been effectively integrated into drug delivery systems, showcasing significant efficacy in targeted treatments for tumours, glaucoma, and certain bacterial infections [35]. Metal‐based nanoparticles can be fabricated using either destructive or constructive synthesis approaches. Commonly utilized metals for nanoparticle production include aluminium (Al), cadmium (Cd), cobalt (Co), copper (Cu), gold (Au), iron (Fe), lead (Pb), silver (Ag), and zinc (Zn). Due to their pronounced quantum confinement effects and high surface‐to‐volume ratios, metal nanoparticles demonstrate remarkable ultraviolet–visible sensitivity in addition to exceptional electrical, catalytic, thermal, and antibacterial characteristics [35, 41]. Metal oxide nanoparticles consist of positively charged metal cations and negatively charged oxygen anions. Frequently synthesized examples include silicon dioxide (SiO_2_), titanium dioxide (TiO_2_), zinc oxide (ZnO), and aluminium oxide (Al_2_O_3_). Due to their remarkable optical properties, metal nanomaterials find extensive applications across diverse research and diagnostic applications [38, 41].
3. *Carbon‐based nanomaterials*. Carbon‐based nanomaterials such as fullerenes, graphene derivatives, carbon nanotubes, and quantum dots have exceptional thermal stability, electrical conductivity, and mechanical strength. On top of that, their structural versatility enables extensive biomedical applications, especially in targeted drug delivery, tissue scaffolding, and biosensing [42, 43].

### 2.2 Synthesis of Nanomaterials

**FIGURE 3:**
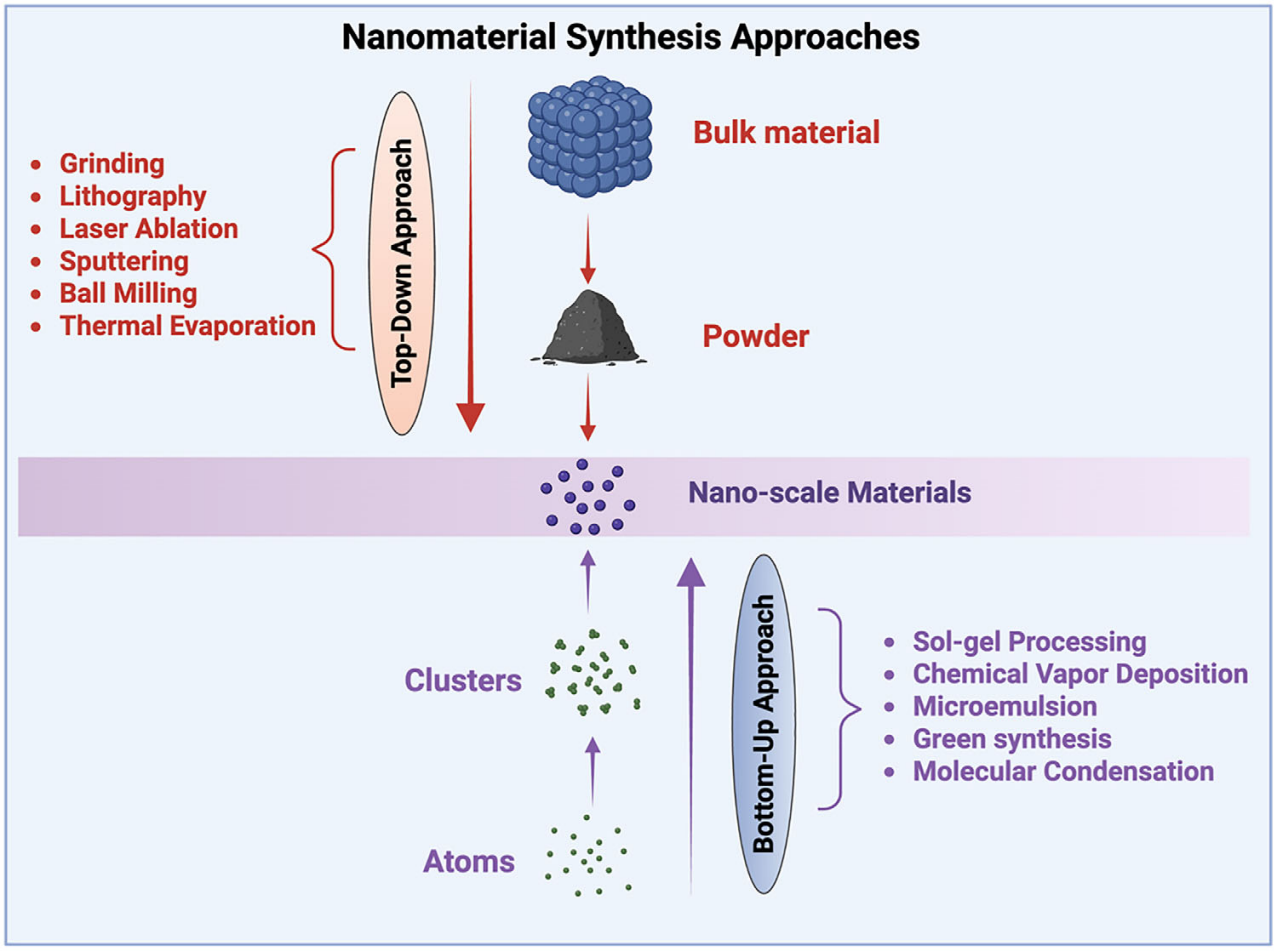
Top‐down and bottom‐up approaches for the synthesis of nanomaterials.

Generally, there are mainly two approaches for synthesizing nanomaterials (Figure 3). Top‐down approach is a method for deconstructing or downsizing macro‐crystalline (bulk material) structures while preserving their original integrity, i.e., it moves from the general to the unique. Nanomaterials can be derived from atoms and molecules in a bottom‐up technique, which starts with the specific and moves on to the broad [44, 45]. For biomedicine and allergy applications, bottom‐up methods are often preferred for creating biocompatible nanoparticles with precise control over size and surface properties, although top‐down techniques remain relevant for specific metallic and pharmaceutical nanomaterials.

1. *Top‐down approach*. Top‐down approach utilizes bulk starting materials for the synthesis of nanomaterials, which can be subsequently modified at the surface or structural level during nanostructure fabrication. The precursor is typically a solid, and nanostructures are produced by mechanical size reduction processes, so the overall strategy is inherently subtractive. The approach begins with a structure or pattern defined at the macroscale and progressively reduces its dimensions to the nanoscale through a sequence of processing steps. Representative top‐down techniques include ball milling [46], mask‐based etching, cutting, thermal evaporation, laser ablation [47], sputtering [48], grinding, photolithography, and electron‐beam lithography [49, 50, 51, 52].
2. *Bottom‐up approach*. The bottom‐up approach in nanomaterial synthesis involves the assembly of larger, complex structures from fundamental building blocks (atoms/molecules) through nanoscale physical and chemical processes. The process typically begins with atoms or molecules that nucleate and aggregate into clusters, which subsequently grow into nanostructures via direct atomic or molecular manipulation. This approach encompasses positional assembly, chemical synthesis, and self‐assembly [53]. This paradigm is particularly suited to generating complex structures that reflect thermodynamically driven arrangements of atoms and often yields nanomaterials with reduced defect densities, as well as improved homogeneity and well‐defined short‐ and long‐range order. Representative bottom‐up techniques include chemical vapour deposition (CVD) [54], hydrothermal synthesis [55], sol–gel processing [56], and reverse micellar methods [57]. Bottom‐up methodologies are extensively employed in biomedicine to fabricate biocompatible nanomaterials, such as sol‐gel‐derived mesoporous silica nanoparticles for sustained drug release in cancer therapeutics [58] and AIT [59], alongside hydrothermally synthesized superparamagnetic iron oxide nanoparticles for targeted delivery and MRI contrast enhancement [60]. Within allergy research, self‐assembly‐driven bottom‐up strategies enable the development of polymeric nanoparticles including VLPs, polyesters, liposomes, and chitosan‐based systems that encapsulate allergens to enhance immunotherapy safety, achieving >50% suppression of pro‐inflammatory cytokines (IL‐4, IL‐5) in preclinical evaluations [61, 62], while also facilitating quantum dots and gold nanoparticles as ultrasensitive platforms for allergen detection in diagnostic assays [63, 64].

### 2.3 Characterization of Nanomaterials

Accurate characterization of nanomaterials is crucial for understanding and controlling their properties and behaviour in allergy diagnosis and treatment, as small changes in shape, size, and surface chemistry can greatly influence the biodistribution, immunogenicity, stability, and assay performance [8, 63].

Standard analytic techniques such as transmission electron microscopy (TEM), dynamic light scattering (DLS), zeta potential measurements, and Fourier‐transform infrared spectroscopy (FTIR) are employed to evaluate particle size, shape, surface charge, stability in fluids, aggregation state, and the presence of functional groups [65]. Advanced tools like X‐ray photoelectron spectroscopy (XPS) and thermogravimetric analysis (TGA) further help to verify surface chemistry and the ligand density. These comprehensive analyses are essential to avert undesirable aggregation in physiological media, biocompatibility, and provide regulated interactions with immune cells or allergens [66, 67].

### 2.4 Nano‐Immune Interactions in Allergic Diseases

In allergy diagnostics, advanced nanoparticle surface tuning has improved the precision of nano‐ELISA platforms and nanomaterial‐based biosensors, while nanomaterials also hold substantial promise in allergy treatment by overcoming major drawbacks of traditional soluble allergen AIT — including long treatment duration, limited long‐term efficacy, poor patient compliance, and the risk of systemic adverse events — through highly controlled interactions with the immune system enabled by precise manipulation of four key determinants: size, shape, surface modification, and ligand density [68, 69, 70]. For instance, cationic dendrimers efficiently capture sIgE in nano‐ELISA, while polyethylene glycol (PEG) prevents serum aggregation [71]. Zwitterionic surfaces, on the other hand, optimize stability for point‐of‐care biosensors that can detect peanut or pollen allergens with femtogram sensitivity [64]. These surface modifications improve diagnostic accuracy by boosting sIgE detection sensitivity without triggering unintended hypersensitivity or toxicity [64]. Spherical quantum dots and rod‐like gold nanoparticles also improve cellular uptake in multiplex sIgE assays and BAT flow cytometry, minimizing background noise and improving diagnostic reliability [8, 72].

**FIGURE 4:**
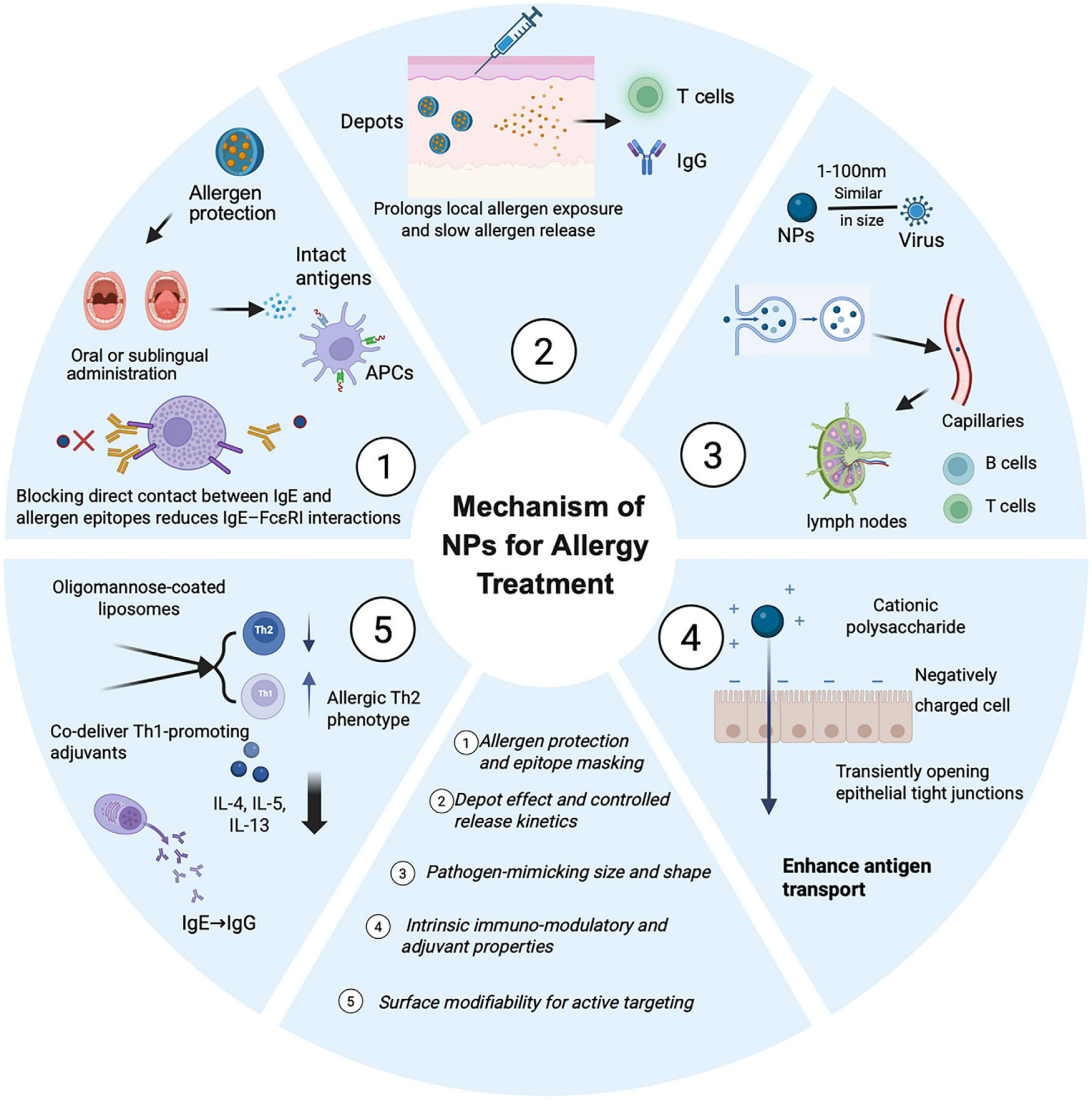
Mechanism of nanomaterials for allergy treatment.

Nanomaterials also hold substantial promise in AIT due to their ability to modulate immune responses. Particle size modulates lymphatic trafficking, endocytic pathway, depot formation, and Fc receptor clustering. Particle shape regulates pattern recognition receptor engagement, cytokine production, and overall safety profile. Surface modification affects mucosal penetration, the cellular uptake pathway, and immune polarization. Ligand density determines epitope spacing and Fc receptor clustering. These determinants collectively confer nanoparticles with unique spatial and temporal control to enhance safety, efficacy, immunomodulation, and targeted delivery as detailed below and in Figure 4.

1. *Allergen protection and spatial control of receptor clustering*. Encapsulation of allergens in nanoparticles such as poly(anhydride) prevents premature degradation of allergens and enhances gut retention to maximize the delivery of intact antigens to antigen‐presenting cells (APCs), which is particularly important for oral and sublingual administration [26, 73]. Moreover, nanoparticles enable precise spatial control of FcεRI receptor clustering through ligand density and particle size. The optimal inter‐receptor distance for maximal IgE‐FcεRI cross‐linking is 20 nm, which initiates downstream Lyn/Syk kinase signaling cascade that drives degranulation in mast cells and basophils [74]. Engineering NPs at low ligand density (43 nm) resulted in lower mast cell degranulation than higher ligand density (6 and 17 nm), and such a parameter overrides valency (antigen copies) [75]. Conversely, ultra‐high ligand density, such as that utilized by virus‐like particles, is another approach to prevent degranulation through steric hindrance and rigid geometry [26]. Beyond ligand density alone, adopting small NPs creates isolated inter‐particle gaps that effectively disrupt the continuous, global signaling required for complete degranulation across the mast cell membrane [74]. Such fine‐tuning of the spatial arrangement of allergens to avoid the degranulation “sweet spot” drastically reduces the allergenicity of NP‐based AIT. This contrasts sharply with traditional AIT, in which soluble allergens present epitopes stochastically at uncontrolled densities, thus posing a high risk of anaphylaxis.
2. *Depot effect and temporal control of antigen presentation*. In traditional AIT, soluble allergens, which are usually smaller than 20 nm, are rapidly absorbed into the bloodstream with minimal lymphatic drainage and are then quickly cleared out of the body through kidney clearance [76]. This not only increases systemic exposure but also demands high and frequent dosing to induce tolerance. The incorporation of NPs into AIT circumvents these issues by enhancing lymphatic drainage and enabling temporal control through the depot effect for sustained antigen release via modulation of particle size. NPs of 20–100 nm (with 30 nm being the most effective) are drained directly to lymph nodes (LNs) and taken up by LN‐resident DCs for rapid tolerance induction [77, 78]. Lymphatic drainage is negligible for NPs larger than 100 nm; instead, they remain at the injection site, forming a local depot that provides sustained antigen release, allowing long‐term tolerance induction. A dual‐action strategy combining NPs of different size categories, such as AP205 capsid VLP displaying IL‐1β (50 nm) with AddaVax (160 nm) [79], achieves both rapid immune engagement and sustained release, resulting in robust, long‐lasting immunity requiring fewer doses and improved patient compliance.
3. *Pathogen‐mimicking trafficking and antigen presentation*. Nanoparticles can be engineered to mimic key features of pathogens, enabling superior trafficking and antigen presentation mainly governed by size, shape, and ligand density. NPs with a rod shape or smaller than 60 nm, mimicking rod‐shaped bacteria or small viruses, respectively, are taken up via caveolin‐mediated endocytosis, which favors MHC‐I cross‐presentation leading to CD8+ T cell activation and Th1 polarization [73]. On the other hand, spherical NPs mimicking spherical viruses are taken up via clathrin‐mediated endocytosis, which favours MHC‐II presentation, leading to CD4+ T cell activation and regulatory T cell (Treg) polarization. VLPs mimicking the native conformation of viruses have multiple favorable properties as AIT vaccine adjuvant. Apart from its optimal size for lymphatic trafficking as mentioned above, its spherical icosahedral geometry also favors CD4+ T cell activation and Treg polarization, as well as ultra‐high ligand density displayed in a repetitive array. This array effectively activates both T cell‐dependent and T cell‐independent B cell activation to produce high‐affinity IgG antibodies [80]. These superior characteristics are more hardly attainable in traditional AIT, in which the soluble allergens are taken up via non‐specific pinocytosis, lack a defined antigen shape, and show poor uptake by antigen‐presenting cells, resulting in weaker T cell and B cell activation.
4. *Intrinsic immune‐modulatory and adjuvant properties. A* majority of the nanomaterials being studied serve not only as carriers but also possess immunomodulatory properties, including polymeric NPs, VLPs, LAMPs, protamine‐based NPs, and chitosan NPs [26]. These nanomaterials mainly promote a Th1 response in comparison to alum, which is a Th2‐promoting adjuvant extensively used in traditional AIT [26, 81]. Non‐immunogenic NPs like liposomes require co‐administration with other NPs or adjuvants to stimulate the immune system. Of note, chitosan NPs derived from crustacean shells are cationic polysaccharides; the positive charge provides intrinsic adjuvant properties through multiple mechanisms [82]. For instance, it facilitates interaction with negatively charged cell membranes, transiently opening epithelial tight junctions to enhance antigen transport, while the mucoadhesive property prolongs antigen retention at mucosal surfaces for sustained immune stimulation. Additionally, chitosan activates the NLRP3 inflammasome via potassium efflux, producing IL‐1β and IL‐18 that support Th1/Treg balance [83].
5. *Targeted delivery and immune polarization*. Traditional AIT delivers soluble allergens non‐specifically into the systemic circulation and local tissues. This often results in off‐target effects, as only a minor portion of the allergen reaches the lymphatic system and leads to suboptimal stimulation of immune cells and the need for higher and more frequent dosing for tolerance induction. Nanomaterial‐based AIT overcomes these limitations by allowing surface modification with targeting ligands to precisely deliver antigen to specific immune cells and direct immune polarization. For instance, oligomannose‐coated liposomes effectively target DCs expressing mannose receptors, inducing IL‐10 production, CD8+ T cell and Treg polarization [84]. Another study designed liver‐targeting lipid nanoparticles decorated with a mannose ligand to bind CD206 on tolerogenic APCs known as liver sinusoidal endothelial cells (LSECs), leading to Treg polarization [85]. Moreover, synthetic glycan ligands for Siglec‐2 (CD22), which is an inhibitory receptor on B cells, were co‐displayed with allergens on the liposomal surface. This enabled a specific immune polarization to suppress allergen‐specific B cell activation, thereby reducing IgE production [86].

## 3 Applications of Nanomaterials in Allergy Diagnosis

Reliable diagnostics require methods that can verify sensitization, reflect clinical allergy, and link immune responses to relevant allergen exposure, particularly for IgE‐mediated reactions triggered by aeroallergens, foods or drugs. However, classical diagnostic platforms – serum sIgE assays and functional BAT and MAT – face persistent challenges. These include suboptimal sensitivity for low‐abundance IgE, cross‐reactivity, limited multiplexing capability, and dependence on specialized laboratory infrastructure [8, 87, 88]. Nanomaterials, including nanoparticles (NPs), quantum dots (QDs), gold NPs (AuNPs), and graphene‐based structures, directly address these issues by offering superior signal amplification, improved specificity, and higher analytical sensitivity, particularly through nano‐biosensors and enhanced functional tests (Table 1) [8, 63, 89].

**TABLE 1:** Representative nanomaterial‐based platforms for serum allergen‐specific IgE (sIgE) detection.

Nanomaterial	Targeted Allergen	Detection Method	Key Performance Improvements	Limitations	Translational Readiness	Reference
Gold Nanoparticle (AuNP) Film	Ara h 2 (Peanut)	Electrochemical	Ultrasensitive detection of low‐abundance IgE	Requires specialized equipment	Low (Proof of concept)	[95]
Silver Nanoparticle (AgNP) Hybrid Probes	Total IgE	Metal‐Enhanced Fluorescence (MEF)	Dramatic signal amplification	Stability & potential toxicity	Low–Moderate (Proof of concept	[96]
AuNP Aggregation Assay	Peanut, Milk, Egg	Colorimetric (Visual)	Naked‐eye readout, simple	Limited specificity in in complex matrices	Moderate (Proof of concept for visual multiplex detection)	[97]
Fe_3_O_4_@SiO_2_ Magnetic Nanoparticles	Can f 1 (Dog dander)	Chemiluminescence	High‐throughput, easy separation	Multi‐step protocol	Moderate (Proof of concept for high‐throughput sIgE assay	[98]
InP/ZnS Quantum Dots (FRET)	Shrimp Arginine Kinase	Fluorescence (FRET)	Cadmium‐free, biocompatible	Complex synthesis	Low (Proof of concept)	[99]
Silica‐coated CdTe Quantum Dots	Bovine β‐lactoglobulin (Milk)	Fluorescent sandwich ELISA (H_2_O_2_ quenching)	∼20‐fold improvement over conventional HRP‐ELISA	Cd‐based toxicity concerns	Low (Proof of concept)	[100]
CdSe/ZnS QD Nanobeads (QB‐FLISA)	Glycinin (Soy)	Fluorescence (QB‐FLISA)	∼7‐fold high sensitivity & multiplexing	Cadmium‐containing QDs raise toxicity issues	Low–Moderate (Proof of concept)	[101]
B/N co‐doped Carbon Dots	Ara h 3 (Peanut)	Dual‐mode (Colorimetric + Fluorescence)	Eco‐friendly, low toxicity	Moderate sensitivity	Low (Proof of concept)	[102]

### 3.1 Nanomaterials in Serum‐Based Allergen‐Specific IgE Detection

The detection of sIgE plays a pivotal role in diagnosing IgE‐mediated allergic diseases by identifying sensitization to inhalant, food or drug allergens [90, 91, 92]. Correlating sIgE levels with clinical symptoms is essential for an accurate diagnosis and guide patient management [93]. Conventional serum sIgE assays, including ImmunoCAP and multiplex platforms, are often constrained by high detection thresholds (detection limits around 0.1‐0.35 kUA/L), cross‐reactivity, and challenges in detecting low‐abundance sIgE within complex serum matrices. These have driven the integration of nanomaterials to enhance analytical performance [93, 94].

**FIGURE 5:**
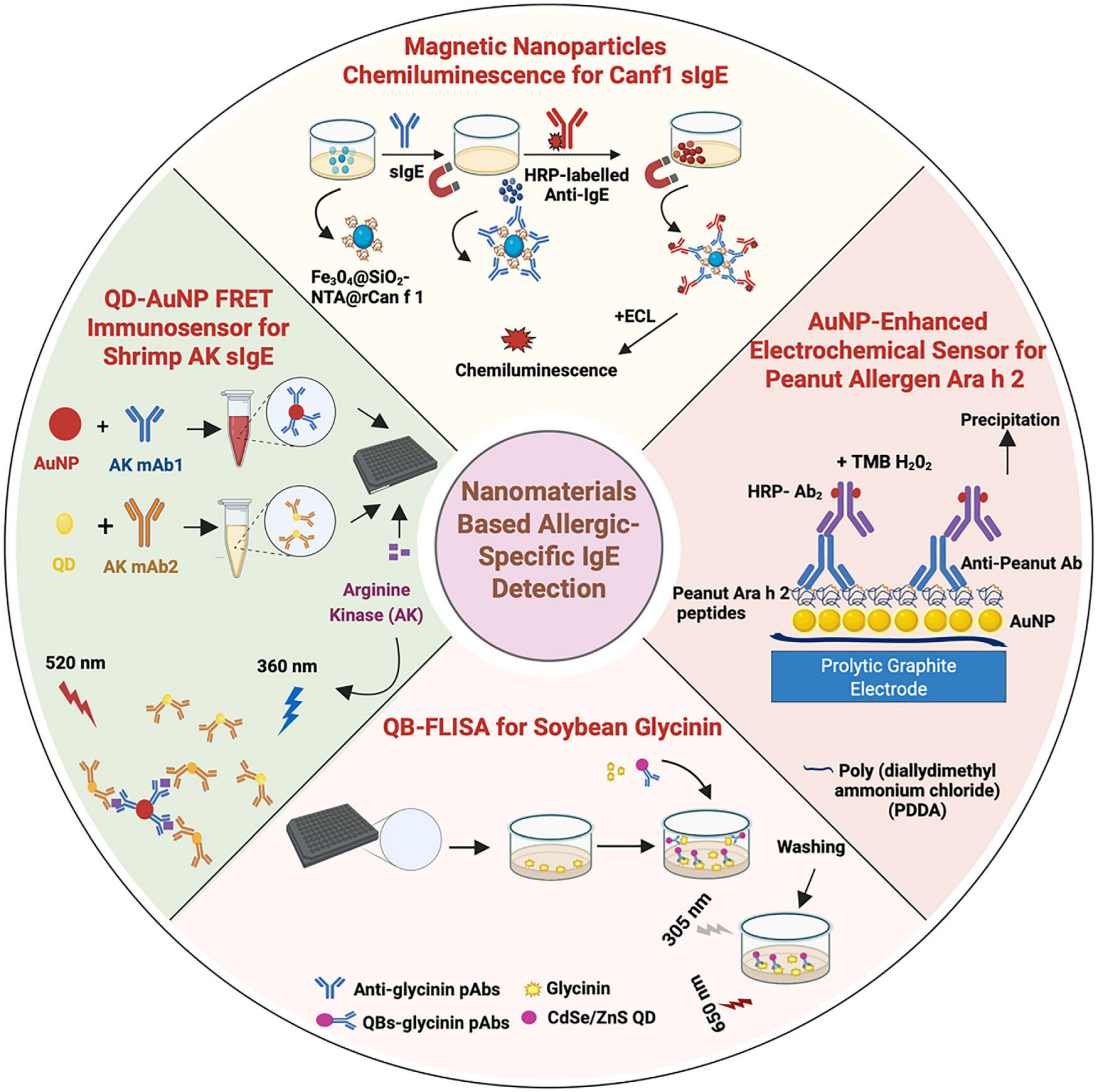
Overview of nanomaterial‐enhanced platforms for allergy diagnosis: Serum‐based detection of allergen‐specific IgE (sIgE) via electrochemical, fluorescence, plasmonic, magnetic, and QD‐based immunoassays.

Early proof‐of‐concept studies in the allergy field used gold nanoparticles (AuNPs) to enhance sIgE detection in peanut allergy. Liu et al. fabricated a nanostructured AuNP film on the electrode surface area that markedly increased the effective surface area and biocompatibility, which in turn promoted efficient immobilization of the Ara h 2 epitope peptide and subsequent capture of patient‐derived sIgE antibodies (Figure 5). In this electrochemical immunosensor, 5 nm glutathione‐capped AuNPs self‐assembled on pyrolytic graphite electrodes, providing a high‐surface, biocompatible interface that enabled oriented presentation of the Ara h 2 peptide and ultrasensitive detection of peanut‐specific sIgE in serum down to femtomolar concentrations via horseradish peroxidase (HRP)‐mediated redox amplification—performance that is particularly valuable for early diagnosis in sensitized individuals with low sIgE levels (<0.35 kUA/L), and typically undetectable by standard clinical tests [95].

Shortly thereafter, fluorescence‐based platforms that incorporated metallic nanostructures rapidly emerged, offering enhanced signal amplification through phenomena like metal‐enhanced fluorescence (MEF). In 2011, a highly sensitive fluorescence assay for the detection of IgE was reported, employing hybrid probes based on silver nanoparticles (AgNPs). In this system, two aptamer‐modified AgNPs, each tagged with Cy3 fluorophores as well as complementary DNA strands, formed aggregates upon binding the target IgE, using MEF to boost the fluorescence signal by up to 100‐fold. This method achieved a detection limit of 0.25 ng/mL IgE, approximately 100 times more sensitive than standard ELISA, highlighting its potential for highly sensitive IgE diagnostics [96].

Furthermore, in 2016, a multiplex visual assay was developed to overcome several drawbacks of conventional SPT and serum immunoassays that are invasive or reliant on specialized equipment. This platform exploits the characteristic plasmonic colour shift of AuNPs that occurs when they aggregate through IgE‐mediated cross‐linking.​ In this approach, AuNPs are conjugated with allergens (e.g., peanut, milk, or egg extracts) and then incubated with a minimal volume of patient serum (5 µL). Addition of a secondary anti‐IgE antibody promotes cross‐linking of the AuNPs, triggering controlled aggregation that alters their localized surface plasmon resonance and produces a visible colour transition from red (dispersed) to blue–purple (aggregated), quantifiable by naked‐eye observation or simple spectrophotometry, providing a direct readout of sensitization severity [97].

Researchers have additionally explored magnetic nanoparticles to facilitate high‐throughput screening in allergy diagnostics. One notable approach involves magnetite (Fe_3_O_4_) core‐silica (SiO_2_) shell nanoparticles functionalized with nickel‐nitrilotriacetic acid (Ni‐NTA) for selective binding of histidine‐tagged recombinant allergens, such as rCan f 1 derived from dog dander (Figure 5). The protocol involves incubating serum samples with these modified magnetic nanoparticles to selectively bind sIgE to the immobilized antigens, followed by the addition of HRP‐conjugated anti‐IgE antibodies. Chemiluminescence signals are then generated through the luminol‐H_2_O_2_ reaction and quantified following magnetic separation of the captured complexes. This system, designed for use in 96‐well plates, permits straightforward multiplexing through simple allergen substitution, delivering a limit of detection (LOD) of 0.35 ng/mL for sIgE within a linear range of 2.52–14.02 ng/mL (R^2^ > 0.99). These characteristics highlight its potential for reliable, large‐scale evaluation of allergic sensitization [98].

Building on metallic nanoparticle platforms, quantum dots (QDs) have emerged as one of the key tools for highly sensitive optical readouts in allergy diagnostics. A notable study in 2021 highlighted recent progress in QD‐enhanced immunoassays, particularly for rapid and sensitive food allergen screening (Figure 5). Focusing on arginine kinase (AK) – an important shrimp allergen associated with severe IgE‐mediated reactions, this study designed a fluorescence resonance energy transfer (FRET) immunosensor featuring biocompatible indium phosphide (InP) core encapsulated by a zinc sulfide (ZnS) shell (InP/ZnS) QDs as the donor fluorophore paired with antibody‐conjugated AuNPs as the acceptor. This innovative FRET configuration provided outstanding sensitivity and specificity for AK detection, because it utilizes cadmium‑free QDs and offers a more environmentally friendly alternative to conventional methods used to assess shrimp allergy risks in food products [99].

QDs are particularly useful in allergy diagnostics because their narrow, tunable emission spectra make it possible to measure multiple allergens at the same time without the spectral overlap that limits many conventional fluorophores. For instance, a fluorescent sandwich ELISA developed for bovine β‑lactoglobulin, a major milk allergen, used silica‐coated cadmium telluride (CdTe) quantum dots whose photoluminescence was quenched by hydrogen peroxide generated by HRP in the antigen–antibody complex. In this format, the locally produced H_2_O_2_ efficiently turned off the QD signal, allowing the assay to reach detection limits about 20‑fold lower than those of a standard HRP colorimetric ELISA [100].

Concurrently, a QD nanobead‐based fluorescence‐linked immunosorbent assay (QB‐FLISA) utilising polymer nanobeads embedded with thousands of CdSe/ZnS QDs was devised for the quantification of glycinin in soy products, attaining sevenfold increased sensitivity and reducing total assay time by approximately one‐third compared with standard ELISA protocols (Figure 5) [63, 101]. More recently, leveraging the specificity of nanobodies for enhanced peanut allergy profiling, Yao et al. developed a boron/nitrogen co‐doped carbon dot‐based dual‐mode (colorimetric/ratiometric fluorescence) immunoassay for Ara h 3‐sIgE, utilizing o‐phenylenediamine (o‐PD) oxidation for quenching‐based readouts on magnetic beads; this eco‐friendly platform achieved an LOD of 6–10 ng/mL and excellent serum recovery, offering a low‐toxicity pathway toward point‐of‐care FA [102]. Collectively, these nanomaterial‐enabled platforms, as summarized in Table 1, have significantly improved sIgE detection from laboratory‐bound, moderate‐sensitivity assays into rapid, ultrasensitive, and potentially point‐of‐care‐compatible tools that are redefining component‐resolved allergy diagnostics.

### 3.2 Nanomaterial‐Enhanced Functional Cellular Tests

**FIGURE 6:**
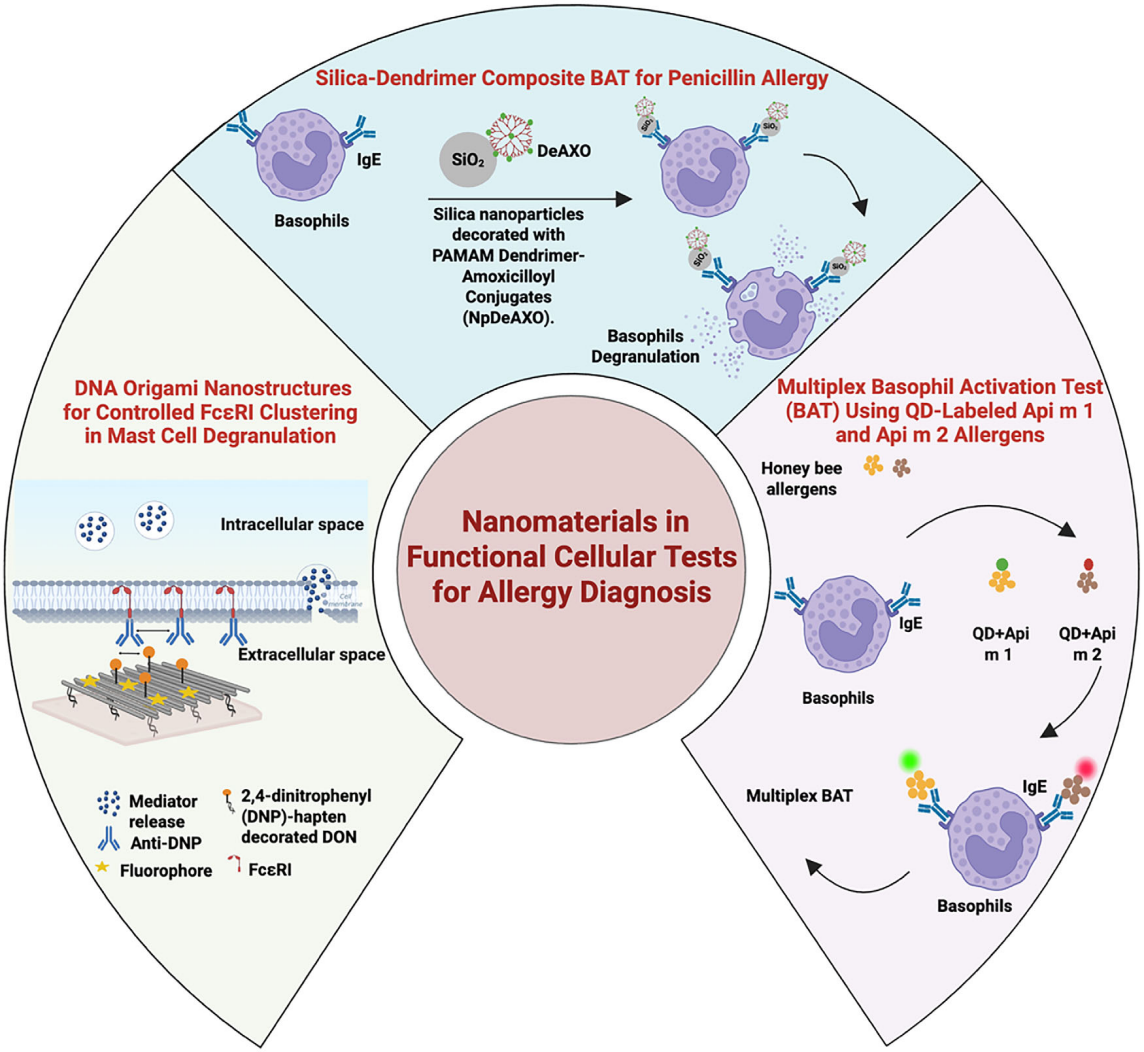
Nanomaterials‐based enhancement of cellular functional assays (BAT and MAT) using quantum dots, dendrimeric‐silica composites, and targeted nanoparticles for multiplexed and sensitive detection of IgE‐mediated responses in allergy diagnostics.

Recent advancements in nanotechnology have revolutionized functional cellular assays like BAT and MAT, mainly by facilitating precise labelling, pronounced signal amplification, and, importantly, multiplex readouts of IgE‐mediated responses on effector cells [8, 75, 103]. QDs have gained particular prominence in BAT due to their bright, stable fluorescence and tunable emission spectra. For instance, amino‐PEG‐coated CdSe/ZnS QDs (emitting at 650 nm) conjugated to major honeybee venom allergens (Api m 1, Api m 2) allow direct staining of surface‐bound sIgE on basophils without requiring secondary antibodies (Figure 6). This greatly improves the accuracy of gating and quantification of activation markers (CD63, CD203c), and achieves multiplex detection in patients. Multiplex BAT protocols have been clinically validated and show strong correlations with phenotypic severity, as evidenced in cetuximab‐induced anaphylaxis, where QD‐labelled allergens predicted grade 4 reactions with 100% sensitivity via elevated CD63 expression (>57% at low concentrations) [104].

In addition to QDs, DNA origami nanostructures (DONs) functionalized with hapten groups like 2,4‐dinitrophenyl (DNP) were also proven effective as customizable platforms for investigating the interactions between sIgE and the high‐affinity FcεRI receptor on mast cells. By arranging bivalent or trivalent DNP ligands at defined intervals (6 to 43 nm) on these rigid scaffolds, IgE‐FcεRI clustering on RBL‐2H3 mast cell lines can be systematically modulated (Figure 6). These experiments showed that mast cell activation peaks when ligands are spaced around 16 nm apart—a distance at which dissociation rates decrease roughly 100‐fold, largely because of enhanced oligovalent binding and favourable receptor diffusion within the plasma membrane. Such findings provide valuable mechanistic insights into features governing FcεRI activation, including intracellular Ca^2^^+^ mobilization and the release of mediators such as β‐hexosaminidase to improve the diagnostic sensitivity of RBL release assays [75].

Meanwhile, silica nanoparticles (SiNPs) hybridized with dendrimeric antigens offer substantial enhancement for BAT for drug allergy diagnosis by mimicking multivalent allergen presentation. In particular, PolyAmidoAmine (PAMAM) dendrimer–silica composites carrying β‐lactam haptens in penicillin‐allergic patients (Figure 6). These particles can activate basophils at much lower allergen doses than soluble drug, leading to roughly a 2–3 fold increase in CD63 expression and improving test sensitivity from about 60% to more than 85%, while cutting down false negative results in low sIgE sera [105]. A concise overview of these nanomaterial‐enhanced cellular assays is provided in Table 2. Taken together, the strategic integration of nanomaterials, including QDs, magnetic particles, dendrimeric composites, and DNA‐based nanosystems has markedly advanced both serum sIgE quantification and functional cell‐based readouts. These developments yield superior sensitivity, improve the physiological relevance of allergy diagnostics, enabling more accurate identification of sensitization patterns and clinical reactivity that mark a significant step forward for more precise and personalized allergology.

**TABLE 2:** Summary of nanomaterials‐enhanced basophil activation tests (BAT) and mast cell activation tests (MAT) for functional assessment of IgE‐mediated allergy.

Nanomaterial	Targeted Allergen	Assay Type	Key Performance Improvement	Key Advantages	Limitations	Translational Readiness	Reference
CdSe/ZnS QDs (Amino‐PEG coated)	Honeybee venom (Api m 1, Api m 2)	BAT	Direct staining, improved gating and multiplexing	Bright fluorescence, no secondary antibody needed	Cadmium toxicity, potential batch variation	Low (Proof of concept)	[104]
DNA Origami Nanostructures (DONs)	Anti‐DNP IgE (hapten‐specific	MAT(RBL‐2H3)	Precise control of epitope spacing	Excellent for studying receptor clustering	Complex design, not suitable for routine diagnostics	Very Low (Proof of concept for mechanistic studies)	[75]
PAMAM Dendrimer–Silica Nanoparticles	Penicillin allergy (β‐lactam haptens‐specific sIgE)	BAT	Enhanced sensitivity (60% → 85%)	Better multivalent presentation	Manufacturing complexity, limited allergen range	Low–Moderate (Proof of concept)	[105]

### 3.3 Comparative Analysis Between Nanomaterial‐Based and Conventional Allergy Diagnostics

Although nanomaterial‐based allergy diagnostics have expanded the analytical capabilities of allergy diagnosis, their clinical value becomes apparent when compared with conventional diagnostic methods. Conventional allergy diagnostic platforms such as singleplex or multiplex serum sIgE immunoassays (e.g., ImmunoCAP), SPT, CRD, BAT, and MAT remain the cornerstone of clinical practice [22, 106, 107, 108, 109]. These methods are widely available, well standardized, and deeply integrated into routine diagnostic workflows. However, they have notable limitations, including modest analytical sensitivity for low‐abundance IgE, frequent cross‐reactivity, and poor correlation between sensitization and actual clinical reactivity [106]. In food allergy, OFC continues to serve as the gold standard for confirming true clinical allergy [110].

From a technical perspective, nanomaterial‐based allergy diagnostics often provide lower detection limits, stronger signal amplification, and better multiplexing capability than conventional immunoassays. However, each nanomaterial class offers a different balance between performance and practicality. AuNPs, for instance, are widely adopted in colourimetric lateral flow assays and electrochemical sensors. Their good biocompatibility, ease of surface modification, and strong plasmonic properties make them well‐suited for visual aggregation tests and electrode‐based detection systems [95, 97]. Silver nanoparticle‐based diagnostics, by contrast, are particularly effective in metal‐enhanced fluorescence (MEF) and often produce marked signal amplification and very low detection limits [96]. QDs are particularly valuable when multiplex detection is required. Their narrow emission spectra, tunable fluorescence, high brightness, and strong photostability make it possible to measure several allergens simultaneously with minimal signal overlap [99, 100, 101]. Magnetic nanoparticles, most commonly Fe_3_O_4_@SiO_2_ core–shell particles, offer practical advantages in sample handling by enabling rapid magnetic separation, supporting high‐throughput 96‐well workflows, and reducing interference from complex biological matrices [98]. Hybrid nanostructures, including QD‐AuNP FRET pairs and dendrimer‐silica composites, combine complementary properties to further improve sensitivity and specificity. In general, hybrid and core‐shell designs tend to perform better than single‐material systems, but may involve trade‐offs in cost, stability and biocompatibility [99, 105]. Dendrimeric and DNA‐origami‐based platforms, in contrast, are especially useful for mechanistic studies because they can present allergen or hapten epitopes in a highly controlled multivalent format that mimics biologically relevant receptor clustering [75].

Despite these remarkable gains in analytical performance, most of the allergy diagnostic platforms based on nanomaterials are still in the preclinical stage or in the early stages of clinical validation. Among them, gold nanoparticle‐ and magnetic nanoparticle‐based tests appear to be closer to practical applications largely because their reliance on relatively simple detection methods such as colourimetry, chemiluminescence, or standard plate‐based readouts allows integration into established laboratory operations [97, 98]. QD‐based systems have excellent analytical sensitivity and multiplexing potential, but larger translational challenges. Hurdles such as assay standardization, concerns about the safety of the materials, and possible nanotoxicity (especially with cadmium‐containing QDs) have hindered their wider real‐world clinical validation beyond small‐scale proof‐of‐concept studies [63]. Other experimental platforms, such as dendrimeric antigens or DNA origami nanostructures offer attractive alternatives for mechanistic research. However, they are still in the early stages of development, with concerns regarding manufacturing scalability, batch‐to‐batch reproducibility, and regulatory constraints needing to be fully solved before clinical translation can be seriously contemplated [75].

In terms of clinical utility, the main value of these nanomaterial‐based allergy diagnostic platforms lies not in replacing established allergy diagnostics, but in complementing them. Conventional sIgE assays, SPT, BAT, and OFC continue to define the clinical framework [111], and these nanomaterial‐based allergy diagnostic platforms must ultimately demonstrate that they improve clinical decision‐making, rather than simply providing superior analytical performance through improving assay sensitivity. Nanomaterial‐based allergy diagnostic platforms hold considerable promise; their superior sensitivity may enable better detection of clinically relevant low‐abundance or high‐affinity IgE, potentially reducing the number of unnecessary OFCs and improving risk stratification. However, direct head‐to‐head studies systematically comparing nanomaterial‐based diagnostic systems with conventional tests and OFC outcomes are still scarce. Existing data are largely limited to analytical validation rather than clinical utility metrics such as positive/negative predictive values, likelihood ratios, or impact on patient management. Larger prospective studies are urgently needed to evaluate whether these analytical improvements may be translated into considerable advances in diagnostic accuracy to reduce healthcare burden and improve patient care [8, 112].

In summary, nanomaterial‐based allergy diagnostics offer clear benefits over traditional allergy diagnostic methods in terms of analytical sensitivity, assay speed, and potential accessibility. However, bridging the gap from bench to bedside will require rigorous standardization, safety evaluation, large‐scale validation against OFC outcomes, and resolution of remaining translational barriers. Importantly, analytical excellence alone is insufficient for clinical adoption. Future nanomaterial‐based platforms must also demonstrate reproducibility, cost‐effectiveness, regulatory feasibility, and meaningful improvements in clinical decision‐making before they can be integrated into routine allergy practice.

## 4 Application of Nanomaterials in Allergy Treatment

The integration of nanomaterials into AIT offers innovative strategies for overcoming the major drawbacks of conventional treatments, such as treatment duration, inconsistent/limited long‐term efficacy, poor patient adherence, and the risk of systemic adverse events [68, 69]. Unlike free allergens or traditional adjuvants, nanomaterials interact with the immune system in highly controlled ways determined by their size, morphology, surface chemistry, and cargo composition [70]. These properties confer unique immunoregulatory capacities upon NPs, enhancing allergen protection, stability, immunomodulation, and targeted delivery.

Although different nanomaterials have been explored for allergy treatment, this review specifically focuses on three major therapeutic platforms: virus‐like particles (VLPs), Poly(lactic‐co‐glycolic acid) (PLGA), and lipid nanoparticles (LNPs). This emphasis reflects the current state of translational development and is guided primarily by three considerations: biocompatibility and safety, regulatory feasibility, and degree of preclinical or clinical advancement. Compared with many inorganic and carbon‐based nanomaterials, VLPs, PLGA, and LNPs possess more favorable biodegradability and better‐defined metabolic clearance pathways. For example, PLGA first degrades into lactic acid and glycolic acid, which are then metabolized into CO_2_ and H_2_O [113], which effectively minimize chronic immunotoxicity [114, 115]. In contrast, several inorganic and carbon‐based nanomaterials continue to face important translational barriers related to long‐term tissue accumulation, oxidative stress, and uncertainties about chronic immunotoxicity. For instance, nanomaterials such as AuNPs and AgNPs have demonstrated exacerbation of Th2 allergic models when exposed in conjunction with Th2‐type allergens like ovalbumin [116]. Pulmonary exposure to AgNPs has been shown to induce an inflammatory response and elevate serum ovalbumin‐specific IgE levels in experimental models [117]. Furthermore, VLPs, PLGA systems, and LNPs are supported by substantially stronger regulatory and manufacturing precedents, as related formulations and materials have already been approved or clinically validated by regulatory bodies such as the Food and Drug Administration (FDA) and European Medicines Agency (EMA) for other clinical applications [118]. Therefore, focusing on these three platforms maintains the clinical and translational relevance of the therapeutic discussion by highlighting the nanomaterial systems currently closest to practical implementation in allergy immunotherapy.

### 4.1 Virus‐Like Particles

Virus‐like particles are highly organized nanostructures formed by the self‐assembly of viral capsid or envelope proteins. Crucially, they lack viral genetic material, rendering them non‐infectious and safe for clinical application [119, 120]. Their structural mimicry of native viruses, with a typical size range of 10–100 nm, enables efficient drainage into lymph nodes and robust uptake by APCs. In the context of AIT, VLPs serve as versatile platforms that can display dense, repetitive arrays of allergens on their surface or encapsulate immunomodulators like CpG motifs to guide immune deviation [119, 121]. This highly ordered, repetitive structure effectively cross‐links B‐cell receptors, inducing robust antibody responses even without conventional adjuvants. Furthermore, VLP‐based vaccines can modulate the immune system by shifting the response away from a Th2‐biased allergic profile to a protective Th1 profile and inducing high‐affinity IgG antibodies that compete with IgE, thereby blocking allergen‐induced effector cell activation (Figure 1).

Peanut allergy is one of the most severe and persistent food allergies, and VLP‐based technology has shown strong promise in preclinical models (Table 3) [122]. Research has focused on coupling major peanut allergens, such as Ara h 1, Ara h 2, or whole roasted peanut extracts, to immunogenic carriers like the Cucumber Mosaic Virus integrated with universal T‐cell epitopes (CuMV_TT_) [123, 124, 125, 126]. Preclinical studies indicate that these VLP‐displayed allergens were highly immunogenic, inducing potent production of allergen‐specific IgG antibodies that intercept allergens before they can cross‐link IgE on mast cells and basophils. Importantly, due to the rigid geometric arrangement of allergens on the VLP surface, these candidates exhibit a reduced capacity to cross‐link FcεRI receptors, thereby minimizing the risk of systemic anaphylaxis during treatment while effectively protecting against anaphylactic challenges in mouse models [122].

**TABLE 3:** Representative nanomaterial‐based platforms for allergen‐specific immunotherapy.

Nanomaterial	Targeted Allergen	Clinical Stage	Route of Administration	Key Immunological Effect	Major Outcome / Safety	Limitations	Translational Readiness	Reference
CuMV_TT_ VLP(Chemical Coupling)	Ara h 1, Ara h 2, or Whole peanut Extract (Ara R)	Preclinical (Mice)	subcutaneous	Induces blocking IgG; engages inhibitory FcyRIIb	Prevents systemic anaphylaxis in mice; hypoallergenic in vitro	Manufacturing complexity; risk of trace “free” allergens remaining	Low‐Medium (Proof of concept)	[123]
CuMV_TT_ VLP(Genetic Fusion / Mosaic VLP Peanut)	Ara h 2	Clinical Phase 1(PROTECT trial)	subcutaneous & SPT	Shifts Th2 to Th1 profile; upregulates DC1; induces IL‐10+ Bregs; blocks IgE binding	Therapeutic and prophylactic protection in mice; zero free allergen; tolerated in 6 allergic human adults	Initial human data relies on a small sample size (n = 6).	High (GMP scaled, active human trials)	[124, 126]
Bivalent AP205 VLP (VLP‐PD1N / VLP‐D2)	Der p 1, Der p 2 (House Dust Mite)	Preclinical (Mice)	Intramuscular	Reduces IL5 and Der p–specific IgE; increases IFNγ to support Th1; induces strong IgG1/IgG2a	Reduced pulmonary inflammation, lowering lung eosinophils and mucus‐producing cells	IgGindependent inflammation reduction; control VLP effective; requires long‐term models; needs TLR7‐deficient mice to separate RNA effects	Low‐Medium (Proof of concept)	[128]
AP205 VLP (SpyCatcher/SpyTag)	Pen m 1 (Shrimp tropomyosin from Penaeus monodon)	Preclinical (Mice and in vitro)	Intramuscular	High‐avidity blocking IgG1/IgG2a induction; Th1‐biased response via IFNγ and particle‐packaged bacterial RNA acting as a TLR7/8 ligand	Hypoallergenic; 41–100× lower effector cell degranulation compared to soluble ST‐Pen m 1.	In vivo protection against shrimp‐induced anaphylaxis has not yet been evaluated.	Low‐Medium (Proof of concept for primary prevention)	[129]
PLGA‐encapsulated MSC‐Exos	OVA (Allergic Rhinitis)	Preclinical (Mice and in vitro)	Intranasal	Boosts Th1/Tregs while suppressing Th2 (IL4), Th17, and IgE; multiomics implicate PPAR and glycolysis pathways	Sustained local release; reduced nasal mucosal immune cell infiltration	OVAonly efficacy; exosome safety unresolved	Low‐Medium (Proof of concept for biomaterial‐facilitated local exosome delivery)	[133]
Allergen‐encapsulating PLGA NPs	OVA	Preclinical (Mice)	Intravenous	Targets pro‐tolerogenic APCs; induces gut‐localized suppressive Tregs; reprograms pathogenic Th2 cells	Directly suppresses pathogenic Th2 responses and reactivity, providing long‐lasting disease suppression of food allergy	The intravenous route limits routine use	Low‐Medium (Preclinical therapeutic proof of concept)	[134]
Spleen‐Targeted Tolerogenic mRNA‐LNPs (sLNP‐OVA/Cel)	OVA(Allergic Asthma)	Preclinical (Mice)	Intravenous	Tolerogenic LNPs via NFκB suppression; splenic DC‐targeted Treg induction; Treg trafficking to the lung to inhibit Th2	Therapeutic & prophylactic efficacy (↓eosinophils, ↓mucus); excellent biocompatibility with no organ toxicity	OVA only; spleen targeting mechanism unresolved	Low‐Medium (Proof of concept for a tolerogenic mRNA‐LNP delivery platform)	[138]
Allergen‐encoding mRNA‐LNPs	Experimental Allergy: Allergen‐encoded	Preclinical (Mice)	Intramuscular	Modulates Tcell differentiation; strongly inhibits pathogenic Th2/Th17 generation	Effective in both the prevention and active treatment of allergic responses	Requires translation from murine models to human clinical application	Low‐Medium (Proof of concept for allergen‐encoded mRNA‐LNP efficacy)	[137]

House dust mite (HDM) allergy is a major driver of asthma and rhinitis, and VLP‐based approaches attempt to overcome the limitations of crude extracts, which are difficult to standardize in AIT [119]. In this domain, strategies often involve coupling major allergens, such as Der p 1 or Der p 2, to viral capsids to boost immunogenicity [127]. The VLP platform is particularly advantageous for HDM allergy as it facilitates the co‐delivery of innate immune response modulators. The latest research has developed a bivalent vaccine based on AP205 bacteriophage‐derived VLPs (VLP‐PD1N/VLP‐D2) for the treatment of HDM‐induced allergic airway inflammation. In mouse models, this platform systemically redirected the Th2‐type immune response toward a Th1‐dominant response while inducing high levels of allergen‐specific blocking antibodies, thereby significantly attenuating pulmonary inflammation and airway symptoms [128].

Shrimp allergy, primarily mediated by the muscle protein tropomyosin, is a prevalent FA with no specific cure, making it a prime candidate for VLP nanovaccine development. VLPs conjugated with shrimp tropomyosin allergen Pen m 1 via SpyTag/SpyCatcher technology exhibited significantly reduced allergenicity but retained high immunogenicity. Preclinical studies report that unadjuvanted VLP‐Pen m 1 induced robust Th1‐biased responses and high titers of Pen m 1‐specific IgG antibodies with strong blocking capacity, inhibiting IgE binding to Pen m 1 by up to 70% [129], thus supporting the potential of VLP‐based platforms as safer and more effective therapeutic candidates for shrimp allergy.

Beyond targeting specific allergens, the unadjuvanted CuMV_TT_ VLP platform, which displays mouse IgE‐Fc fragment domains 3 and 4, provides a universal anti‐IgE immunotherapy. It induces anti‐IgE IgG antibodies that specifically target conserved mannose glycans on IgE. Because this mannose region is essential for FcϵRI binding but hidden on mast cell‐bound IgE, the vaccine safely neutralizes free IgE without cross‐linking receptor‐bound IgE, thus minimizing anaphylaxis risks similar to omalizumab [130].

### 4.2 Poly(lactic‐co‐glycolic acid)

Poly(lactic‐co‐glycolic acid) is an FDA‐approved biocompatible and biodegradable polymer that has been widely applied in drug delivery systems for AIT. Its core advantages lie in the ability to tune polymer properties to obtain ideal antigen release profiles, protecting encapsulated allergens or therapeutic agents from enzymatic degradation (such as digestive enzymes or harsh pH environments), and facilitating antigen uptake and presentation by immune cells such as DCs (Figure 1) [131]. In intranasal immunotherapy, PLGA nanoparticles can not only avoid the hepatic first‐pass metabolism effect, but also overcome mucosal physiological barriers to achieve long‐term clinical tolerance [132]. In a mouse model of allergic rhinitis using PLGA nanoparticles to co‐encapsulate allergens OVA and curcumin for SLIT, the immunomodulatory effect was found to be superior to treatment with the allergen or curcumin alone [131]. Recent research has developed PLGA micro/sub‐micro particles to encapsulate mesenchymal stem cell‐derived exosomes (MSC‐Exos), which significantly alleviated the allergic airway inflammation in rhinitis models [133]. Allergen‐loaded PLGA nanoparticles safely suppress food allergy by evading IgE activation. Two intravenous doses were sufficient to engage tolerogenic DCs to reprogram Th2 cells, expand Foxp3^+^ Tregs in the gut and CD73^+^FR4^+^ anergic T cells, which markedly reduced effector cell degranulation and anaphylaxis [134]. PLGA and its derivatives demonstrate remarkable efficacy across various administration routes in AIT due to their excellent biocompatibility and modifiability. Regarding EPIT, PLGA‐PEG‐PLGA copolymers formulated as nanoparticles and combined with iontophoresis have been confirmed to penetrate skin hair follicles more effectively than ordinary chitosan‐modified or plain PLGA nanoparticles, significantly enhancing IgG1 antibody titers and lowering IgE levels [135]. For SLIT, aptamer‐modified PLGA nanoparticles (Apt‐OVA‐PLGA) have been developed to target DCs; this system significantly inhibited IgE and Th2‐type cytokines (IL‐4, IL‐17a) at lower antigen doses, while enhancing Th1 and Treg responses by upregulating IFN‐γ, IL‐10, and TGF‐β levels, effectively restoring allergic immune imbalance and supporting long‐term tolerance induction [136].

### 4.3 Lipid Nanoparticle

In recent years, mRNA‐lipid nanoparticle (mRNA‐LNP) technology has demonstrated potential in allergy immunotherapy. In mice asthma models, mRNA‐LNP vaccination effectively suppressed Th2/Th17 responses, reduced IL‐4, IL‐5, and IL‐13, while enhancing Th1 and CD8+ T cell activity and promoting IFN‐γ production. Importantly, it increased allergen‐specific IgG levels while maintaining low IgE, thereby blocking IgE‐mediated allergic reactions. Animal studies revealed significant reductions in eosinophil infiltration and airway mucus secretion, improved airway hyperresponsiveness in both acute and chronic asthma [137]. Wang et al. recently developed a spleen‐targeted tolerogenic mRNA–LNP vaccine that co‐delivered allergen‐encoding mRNA and the anti‐inflammatory agent celastrol to DCs. This approach induced tolerogenic DCs, antigen‐specific Tregs and suppressed Th2‐driven inflammation in an asthma model. Two intravenous doses significantly reduced airway eosinophilia, mucus secretion, and IgE levels, demonstrating strong prophylactic and therapeutic effects [138]. However, recent findings indicate that mRNA‐LNPs may exacerbate inflammation under pre‐existing inflammatory conditions, characterized by sharp increases in IL‐6 and MIP‐2, involving macrophages and TLR4 signaling. Thus, careful evaluation of patients’ inflammatory status is essential before clinical application [139].

Beyond mRNA, LNPs can be surface‐engineered for delivering small molecules directly to the nasal mucosa. Recently, Li et al. designed phosphatidylserine‐containing anionic LNPs to encapsulate bryostatin‐1 for allergic rhinitis immunotherapy. While traditional cationic formulations suffer from mucin entrapment and cellular toxicity, these engineered anionic LNPs successfully bypass mucus trapping, extend nasal retention (>8 h), and optimize B‐cell targeting. Remarkably, even at an ultra‐low dose (0.5 ng), this nano‐formulation effectively drove selective class switch recombination, robustly boosted protective IgA responses while downregulating systemic IgE antibody [140]. This strategy establishes a cost‐effective, stable, and non‐invasive platform for mucosal allergy management. Overall, these findings position LNP‐based platforms as compelling next‐generation AIT alternatives to traditional AIT approaches.

Furthermore, an allergen‐driven “reverse targeting” strategy introduces a highly precise modality for AIT. Curcumin‑loaded ovalbumin protein nanoparticles (CUR‑OVA NPs) leverage allergen‑dependent recognition to deliver immunomodulatory agents directly to sensitized immune cells to enhance therapeutic precision. In OVA‑induced allergy models, CUR‐OVA NPs effectively reduced IgE/IgG1 levels and suppressed CD4^+^ T‑cell and memory B‑cell responses, outperforming free curcumin treatment. This strategy highlights a promising nano‑AIT modality enhancing targeting specificity while attenuating the immune memory responses associated with allergic recurrence. Additionally, the carrier's self‐fluorescence enables real‑time tracking of drug distribution, establishing a highly stable, visible, and specific platform for next‐generation AIT [141].

### 4.4 Mechanistic Advantages and Translational Readiness of Nano‐AIT Platforms

The transition from conventional AIT to nanomaterial‐based AIT represents a fundamental paradigm shift from passive allergen exposure to active, rationally designed immunomodulation. While conventional AIT has been the cornerstone of allergy treatment for over a century, its use of free allergens forces a long treatment time (typically 3–5 years) and carries a persistent risk of triggering IgE‐mediated systemic anaphylaxis [24].

Nanomaterials effectively address these limitations. As thoroughly discussed across the VLP, PLGA, and LNP platforms, the success of nano‐AIT relies on two key mechanisms: antigen shielding and targeted immune modulation. Encapsulation or spatial presentation of allergens within nanostructures minimizes effector cell activation. Meanwhile, the pathogen‐mimicking properties of these particles promote rapid uptake by antigen‐presenting cells in the LNs, thereby promoting the Th2‐to‐Th1/Treg immune shift and potentially reducing the treatment duration and dose intensity required for effective AIT.

Among the currently investigated nano‐AIT systems, VLPs appear to possess the strongest translational momentum and the most advanced clinical evaluation. Their superiority in readiness stems from their intrinsically safe, non‐infectious pseudo‐viral architecture and their established clinical track record. Currently, VLP‐based platforms represent one of the most clinically advanced systems under investigation. A VLP platform targeting peanut allergy (CuMV_TT_ displaying Ara h 2) has already entered Phase 1 clinical trials (ClinicalTrials.gov Identifier: NCT05476497) to evaluate its safety and tolerability. Furthermore, other VLP formulations have advanced even further: the CpG‐loaded bacteriophage VLP (QbG10) for house dust mite allergy has completed Phase 2b trials (NCT00800332), and the BM32 vaccine for grass pollen allergy has reached Phase 2 status (NCT01538979). In the Phase IIb trial of CYT003‐QbG10 for house dust mite allergy, through the safe co‐delivery of an innate immune modulator, this platform achieved a 10‐fold increase in clinical allergen tolerance, alongside significant improvements in patient quality of life [142]. Although the Phase II trial of the BM32 grass pollen vaccine did not reach its primary clinical endpoint, its success in selectively inducing protective IgG without boosting pathogenic IgE provides a crucial preliminary demonstration of the inherent safety of carrier‐based spatial shielding [143]. Previous literature often attributes VLP limitations primarily to anti‐carrier immune responses. However, a more important translational challenge may lie in the complex chemistry, manufacturing, and control (CMC) requirements required to maintain highly uniform and repetitive antigen display without disrupting particle assembly [80]. To address these specific manufacturing hurdles, modular “plug‐and‐display” technologies like the SpyCatcher/SpyTag system offer a robust solution by ensuring consistent antigen conjugation and bypassing the steric and size limitations of traditional genetic fusion, thereby facilitating rapid and scalable vaccine generation [80]. Ultimately, given their active clinical status and these emerging manufacturing solutions, VLPs remain the highest priority for AIT translation.

Both PLGA and LNPs occupy a low‐to‐medium translational readiness. PLGA is extensively investigated and benefits from being an FDA‐approved, biocompatible polymer with highly scalable manufacturing protocols [131, 136]. While PLGA‐based AIT successfully transitioned into human trials with the peanut allergy candidate vaccine CNP‐201 (NCT04950504, NCT05250856), the recent discontinuation of these studies highlights the steep and often volatile barriers to clinical translation. Other PLGA platforms remain largely confined to preclinical models, demonstrating efficacy primarily in mouse models of peanut and respiratory allergies [144] Indeed, the broader application of PLGA in AIT still faces limitations such as particle aggregation, complex manufacturing, and poor encapsulation efficiency for hydrophilic allergens [135, 145] Future advancements can focus on developing thermosensitive hydrogels or PEG‐modified copolymers to simplify sterilization and prevent aggregation, alongside optimizing non‐invasive local administration routes to enhance targeted immune responses [132]. The manufacturing of LNPs is mature post‐COVID‐19 [146]. However, its application in allergy is currently restricted to preclinical proof‐of‐concept studies, such as the delivery of mRNA‐encoded Ara h 2 epitopes to the liver or spleen in murine models [85, 138]. The major limitation hindering the clinical trial readiness of LNPs for AIT is their inherent adjuvanticity. The ionizable lipids and PEGylated components essential for LNPs can trigger robust innate immune and inflammatory responses [140, 146]. Because AIT requires the induction of immune tolerance rather than inflammation, its formulations require further immunological fine‐tuning to consistently induce tolerance rather than inflammatory sensitization in food allergy contexts.

### 4.5 Safety and Immunotoxicological Considerations of Nano‐AIT Platforms

Despite the considerable therapeutic promise of nano‐AIT, safety and immunotoxicological considerations remain major translational challenges. For VLP‐based systems, current evidence generally supports favorable safety profiles, as these particles are non‐infectious and lack replicative viral genetic materials. Clinical studies involving VLP‐based allergy vaccines, such as CuMV_TT_‐Ara h 2, have shown acceptable tolerability, with no significant allergic skin reactions observed following SPT administration [124]. PLGA, on the other hand, has already been approved by the FDA for multiple drug delivery formulations given its biocompatibility, physical stability, and processability. Its encapsulation properties also make it favorable for oral and sublingual immunotherapy applications. However, despite its biodegradable profile, PLGA may exhibit prolonged degradation times of up to 24 months, while the initial burst release of the encapsulated allergens may still pose safety concerns, particularly in highly sensitized individuals [61].

As discussed above, nanomaterials interact extensively with the immune system, influencing complement activation, cytokine release, APC uptake, and biodistribution. Certain nanostructures may unintentionally activate innate immune pathways, including macrophages and Toll‐like receptor signaling, thereby aggravating allergic airway inflammation [116, 117, 139, 146]. Another important but often under‐discussed issue involves the interaction of nanomaterials with the epithelial and mucosal barriers, where enhanced barrier penetration may paradoxically promote sensitization, local inflammation or increased allergen uptake [116]. These findings collectively highlight that some nanomaterials may shift the immune response toward inflammatory sensitization rather than tolerance. Therefore, careful evaluation of the physicochemical properties of nanomaterials remains essential for the safe clinical translation of nano‐AIT platforms.

## 5 Future Directions

Despite the significant advances in the application of nanomaterials for allergy diagnosis and treatment, their journey from bench to bedside is impeded by a formidable array of clinical translation hindrances. Moving forward, a structured and interdisciplinary approach is essential to overcome the existing barriers. Below, we outline several key areas that require focused research efforts, along with practical directions for future development.

As discussed, a primary concern revolves around the safety and immunotoxicity of nanomaterials, especially given that allergic individuals already have a dysregulated immune system. Although existing evidence suggests that selected nanoplatforms possess acceptable preliminary safety profiles, most available data are derived from short‐term animal models or early‐phase clinical trials; comprehensive long‐term toxicological data in allergic models and human populations remain limited. Future development will also require more standardized and allergy‐specific immunotoxicological evaluation frameworks incorporating long‐term biodistribution studies and complement activation testing. Importantly, promising in vitro and animal study results often fail to translate effectively into human clinical efficacy due to the inherent complexity of human immunobiology. Particular attention must be directed toward mast cell and basophil degranulation, Th2 skewing, nanoparticle‐induced inflammation, and epithelial barrier disruption. The development of safer, biodegradable, and cadmium‐free nanomaterials (e.g., carbon dots, InP/ZnS QDs, and fully organic nanoparticles) should be further accelerated. Standardized preclinical safety testing protocols specifically designed for allergic populations are urgently needed.

A second critical barrier is the challenge of reproducibility, scalable manufacturing, and standardization. Minute variations in size, shape, surface charge, and batch composition can drastically alter the biological activity, biodistribution, and immune interactions of nanomaterials. Ensuring consistent batch‐to‐batch production, particularly during industrial scale‐up, is essential but technically demanding. Future efforts should therefore prioritize precise control of the physicochemical properties of nanomaterials under Good Manufacturing Practice (GMP) conditions, alongside the development of robust characterization standards and validated assays for evaluating the functional performance and stability of nanomaterials.

The regulatory landscape for nanomedicine also remains insufficiently developed. Existing regulatory frameworks are largely designed for conventional drugs or medical devices and often do not adequately accommodate the unique physicochemical and immunological properties of nanomaterials. Consequently, approval pathways can be ambiguous, lengthy, and costly, that create skepticism for industrial investment and more widespread clinical translation. Greater collaboration among researchers, industrial stakeholders, and regulatory authorities is required to establish clearer, nanomaterial‐specific guidelines, such as standardized classification systems and safety evaluation protocols, and nanomaterial‐specific evaluation criteria to accelerate clinical adoption.

There is also a pressing need for well‐designed translational and clinical studies. Many current studies are still at early stages, based on proof‐of‐concept experiments or small preclinical groups, so emphasizing the importance of larger and more rigorously designed clinical trials. Ideally, these should be multicentre and prospective, with clinically meaningful endpoints. For diagnostics, trials should evaluate not only analytical sensitivity but also clinical utility outcomes, such as reduction in OFC burden, improved risk stratification, and cost‐effectiveness. Future trials on nano‐AIT should incorporate standardized endpoints, such as desensitization thresholds and sustained unresponsiveness determined by double‐blind, placebo‐controlled challenge, and immunological biomarkers such as IgG4 induction. Long‐term follow‐up assessing disease remission, real‐world safety, quality of life improvement, and cost‐effectiveness compared with conventional therapies will be essential for successful clinical translation.

Looking ahead, precision medicine is likely to be one of the most promising future directions for nanomaterials in allergy. Rather than relying on a one‐size‐fits‐all approach, future nanoplatforms will likely need to be tailored to individual sensitization patterns, epitope profiles, and immune phenotypes. In diagnostics, this may involve multiplex platforms capable of profiling component‐level sensitization patterns, low‐abundance IgE, and co‐sensitization signatures. Therapeutically, nanocarriers can be designed to deliver allergens, adjuvants or immune‐modulatory agents in a patient‐specific manner based on disease severity and immunophenotype. Integration of nanotechnology with biomarkers, multi‐omics data and clinical phenotyping may further refine patient selection and improve treatment outcomes. In this context, nanomaterials may act not only as detection and delivery platforms, but also as enabling technologies for advancing personalized allergy medicine.

Overall, overcoming these translational barriers will demand a coordinated interdisciplinary effort among clinicians, immunologists, material scientists, engineers, industrial stakeholders, and regulatory agencies. By systematically addressing safety, manufacturing scalability, regulatory clarity, and clinical validation, nanomaterial‐enabled platforms can facilitate the development of safer, more effective, and increasingly personalized approaches for allergy diagnosis and immunotherapy.

## 6 Conclusions

Allergies remain a significant global health challenge, yet existing diagnostic methods often lack sufficient precision, and current immunotherapies (AIT) continue to face important limitations related to efficacy, treatment duration and safety. Nanomaterial‐enabled platforms have emerged as promising tools for advancing the field of allergology by offering detection platforms with enhanced analytical sensitivity, improved allergen stability and targeted immune modulation. In the therapeutic domain, nanomaterials like VLPs and PLGA‐based systems demonstrate considerable potential for developing safer, more effective, and more personalized AIT strategies. However, as the field advances, several key challenges must be addressed before translating nanomaterials into routine clinical practice. Most nanomaterial‐based allergy platforms remain in the early stages of translational development; long‐term treatment efficacy and safety, immunotoxicity, scalable manufacturing, and standardization remain major hurdles. Addressing these barriers will require coordinated multidisciplinary efforts across academia, industry, and regulatory bodies. Continued advances in nanotechnology and more vigorous clinical validation may ultimately unlock the full diagnostic and therapeutic potential of nanomaterials in allergy medicine.

## Author Contributions

**Madiha Habib**: writing – original draft, writing – review and editing, visualization. **Shan Jiang**: writing – original draft, writing – review and editing, visualization. **Joyce ZX Lee**: writing – original draft, writing – review and editing, visualization. **CW Lim**: writing – original draft. **HL Yeung**: writing – original draft. **Nicki YH Leung**: writing – review and editing. **Patrick SC Leung**: writing – review and editing. **Ting F Leung**: writing – review and editing, funding acquisition, supervision. **Christine YY Wai**: conceptualization, writing – original draft, writing – review and editing, funding acqusition, supervision.

## Funding

This review was funded by the General Research Fund (Reference 14113425) and Research Impact Fund (Reference R4035‐19) of the Research Grants Council and Health and Medical Research Fund (Reference 12230466) of the Health Bureau, Hong Kong SAR Government; and Young Scientist Fund of the National Natural Science Foundation of China (Reference 8250069008). Habib was supported by the Research Committee Postdoctoral Fellowship Scheme (Reference PDFS2024/0716/24jh) of The Chinese University of Hong Kong.

## Conflicts of Interest

The authors declare no conflict of interest.

## Data Availability

Data sharing not applicable to this article as no datasets were generated or analyzed during the current study.
